# Technical note: validation of an automated feeding system for measuring individual animal feed intake in sheep housed in groups

**DOI:** 10.1093/tas/txaa007

**Published:** 2020-01-17

**Authors:** Stephanie K Muir, Nick P Linden, Andrew Kennedy, Grace Calder, Gavin Kearney, Richard Roberts, Matthew I Knight, Ralph Behrendt

**Affiliations:** 1 Agriculture Victoria Research, Department of Jobs, Precincts and Regions, Hamilton, Victoria, Australia; 2 Biosecurity and Agriculture Services, Department of Jobs, Precints and Regions, Rutherglen, Victoria, Australia; 3 Thrive Agri Services, Hamilton, Victoria, Australia; 4 Biosecurity and Agriculture Services, Department of Jobs, Precints and Regions, Ballarat, Victoria, Australia; 5 ZI-Argus Australia Pty Ltd., Clayton, Victoria, Australia

**Keywords:** automated feeding systems, data capture, feed intake, sheep

## Abstract

The development of feeding systems that can individually measure and control feed intake in a group-housed environment would allow a greater understanding of sheep intake without compromising animal welfare and behavior through the removal of social interactions between sheep. This study validated an automated feeding system for measuring feed intake of individual sheep when housed in groups. Validation of the feeding system was conducted during three separate experiments. The validation sampling involved the activation of four individual “feed events,” whereby four separate samples weighing approximately 50, 100, 200, and 400 g were removed from each feeder, with each feed event being linked to a specific radio frequency identification (RFID) tag. The feeder validation experiments evaluated the ability of the feeding system to 1) create a unique feed event every time a sample of pellets was collected from the feeder, 2) link the feed event to the correct RFID, and 3) accurately record the weight of feed that was manually removed. All feed events were initiated and logged in the feeding system with 100% of the events being linked to the correct test RFID. Concordance correlation coefficients between the feeding system-recorded feed weight and the manually removed weight were 0.99 within all three experiments. There was also no overall and little level-dependent bias between the weights measured by the feeding system and weights measured on the external scales. These results indicate the stability of the feeding system over time and consistency between the feeders within and across the three experiments. In conclusion, the automated feeding system developed for measuring individual animal feed intake was able to detect and record the unique electronic RFID associated with unique feed events and accurately capture the weight of feed removed. Furthermore, there was no change in the accuracy of the system from the start to the end of experimental periods, and the amount of feed removed in the feed event (or meal size) did not impact the accuracy of the results.

## INTRODUCTION

Understanding feed intake of sheep has significant implications for nutritional management; however, most data that underpins feeding standards have been collected from sheep that have been individually housed and fed. This is time consuming, labor intensive and expensive, resulting in restrictions on the number of animals that can be measured at a given time. Sheep are highly gregarious and social, exhibiting flocking behavior and require social interactions (Fisher and Matthews, 2001; Hinch, 2017). Sheep exhibit conflict between the need for social interactions and the requirement for food, which can influence food preferences and grazing behavior (Dumont and Boissy, 2000). As a result, individual penning can result in separation anxiety and immune system compromise (Degrabriele and Fell, 2001) and reduce performance and intake, resulting in compromised welfare (Fisher and Matthews, 2001). Feed intake has been shown to reduce when sheep are housed in groups of less than four animals (Penning et al., 1993). Kromann et al. (1971) observed a 10% reduction in daily live weight gain for individually penned lambs when compared to group fed lambs. The ability to express social interactions and feeding behaviors could be important considerations in determining feed intake, maintenance requirements and feed conversion efficiency. However, group feeding of sheep does not allow for the accurate measurement of feed intake in individuals. Malik et al. (1996) grouped lambs of the same genotype in pens of four and calculated intakes on a pen average basis. While this approach did not compromise animal performance, compared to individually penned animals, the quality of feed intake data was limited due to its inability to account for individual animal variation.

There remains a need to assess individual animal feed intake within a group-housed environment. The development of feeding systems that can individually record and control feed intake in a group-housed environment would allow a greater understanding of intake without removing social interactions of sheep. The performance of feeding systems that automatically monitor individual animal feed intake in group-housed pigs (McSweeny, 2001; Faltys et al., 2014), dairy cows (Bach et al., 2004, Chizzotti et al., 2015), and dairy heifers (Williams et al., 2011) have all been previously reported. While a description of units used with beef cattle was provided by Dahlke et al. (2008), they provide no assessment of system performance. Currently, we are not aware of any studies evaluating the performance and accuracy of automated feeding systems for group-housed sheep.

This paper describes the validation of an automated feeding system used for measuring individual animal feed intake in group-housed sheep. We hypothesized that the described automated feeding system would determine individual feed events, including the logging of a feed event and the identity of the animal feeding and that there would be no overall or level-dependent bias between the weight of feed samples collected and measured by the automated feeding system and the weight of the same feed samples measured on an external scale. We also hypothesized that the performance of the system would not vary over time when the feeding system was being used by sheep.

## MATERIALS AND METHODS

### Experimental Conditions

Each of the three validation experiments reported here was conducted during a larger experiment that utilized the automated feeding system to measure feed intake of individual sheep between December 2015 and January 2018 (Muir et al. 2018a, 2018b, 2018c). The feed intake tests using sheep (Table 1) ran for 35–71 d, with 11–24 sheep housed in each pen when offered ad libitum at 1,000 g per meal with no limit on the number of meals able to be consumed. The number of feeders tested during the validation experiments varied based on the number of pens and feeders in use at the time of the experiment. No animals were directly utilized for the collection of data during the feeder validation experiments. The conduct of validation sampling did not interfere with animals mentioned or the protocols of the associated experiment as each sample collection was undertaken over a short period of time (5–10 min per feeder). Sheep were excluded from the feeders (entry gate locked) for the duration of each test.

**Table 1.** Characteristics of feed intake experiments associated with feeder validation experiments

	Experiment 1	Experiment 2	Experiment 3
Year	2015/2016	2016	2017/2018
Feed intake test duration, days	35	43	71
Number of validation test, days	8	3	3
Day of validation test	2, 9, 11, 18, 23, 14, 30, 32	10, 11, 12	1, 57, 71
Number of sheep per pen	24	11–15	18–20
Default meal allowance, g/meal	1,000	1,000	1,000
Number of feeders in use	20	10	16–19
Number of feeders tested	20	10	16–19

It was important to conduct validation experiments during a period of real use by sheep to determine if the feeding system would drift over time through regular usage of the feeder units by the sheep. All animal procedures in the associated experiments were approved by the Department of Jobs, Precincts and Regions, Agriculture Research and Extension Animal Ethics Committee (AEC Approval: 2015-09, 2016-06 and 2017-04) and conducted in accordance with the Australian Code for the Care and Use of Animals for Scientific Purposes (Anon, 2013).

### Feeding System

**Figure 1.**
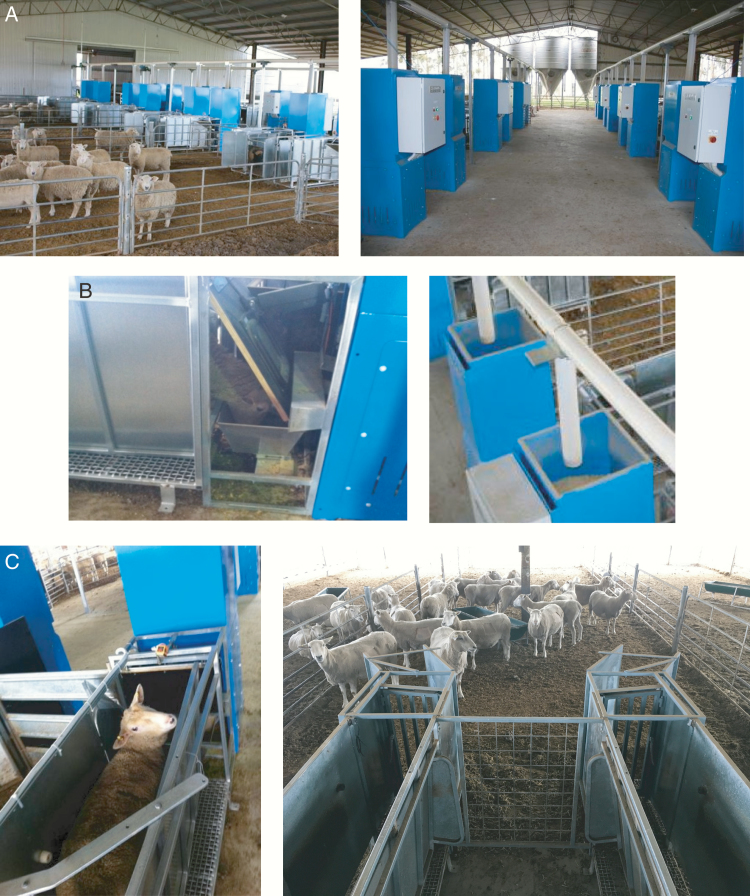
(a) Automated sheep feeding facility showing each pen contains 2 feeders. (b) Storage feed hopper and overhead centerless auger to deliver feed to each hopper and stainless-steel feed bin showing RFID feeder flap and a sheep eating from the feed trough. (c) Approach race configuration showing entry and exit gates and proximity sensor location.

The automated feeding system (Figure 1a) consisted of 20 feeders in 10 pens (2 feeders per pen), which was controlled using Feeder Management Software (FMS). All control processes were managed through the FMS coded in Sequel (SQL—Structured Query Language) by ZI-Argus Australia (Clayton, Victoria, Australia). The FMS software used program logic control to create individual animal feed events under ad libitum feeding conditions and managed data acquisition and storage. Feeding plans for individual animals and the weight of feed to be maintained in the feed bin between feed events were set within the FMS. For ad libitum feeding, feed bins were set to refill between feed events to a feed amount of 1,000 g so that an animal entering the feed station would have access to 1,000 g of feed at each entry. There was no limit on the number of feeder visits (or number of meals) and, hence, the total feed consumption was not restricted.

Each feeder consists of an approach race (Figure 1c) fitted with a photoelectric proximity indicator (Photoelectric sensor 05D100, ifm efector Pty Ltd., Victoria, Australia), an electronically locking gate, a stainless-steel feed bin (total capacity: ~7.5 L) and a larger feed hopper bin (total capacity: ~200 L; Figure 1b). The movement of pelleted feed from the hopper bin to the feed bin is controlled by a centerless auger on a variable speed drive motor. The feed bin is mounted either on two load cells within a steel frame the load cells have a maximum weight capacity of 25 kg, with an accuracy of 0.03% (KPA 6346, Kelba Pty. Ltd., New South Wales, Australia) or a single weighing platform with a maximum weight capacity of 15 kg and an accuracy of 0.03% (PBA226-A, Mettler Toledo Ltd., Victoria, Australia). The proximity sensor detects when an animal is standing in the approach race and activates the creation of a feed event. Animal access to the feed bin is controlled via a plywood flap hinged at the top of the feed bin frame and activated by a 12-V linear actuator (LINAK Pty. Ltd., Victoria, Australia). To capitalize on the proximity of the animal’s head to the plywood flap while waiting to access the feed bin, the radio frequency identification (RFID) reader is mounted on the back of the flap (Figure 1b). The FMS then links the detected RFID to the weight of the feed bin at the start and end of the feed event. The feed event is completed when the photoelectric proximity sensor detects that the animal has left the approach race and the flap closes. The amount of feed consumed in a feed event is calculated by subtracting the end weight from the starting weight of feed in the feed bin. The scales under each feed bin can be calibrated and adjusted to register a zero-tare weight when there is no feed in the feed bin.

### Experiment 1

The first validation experiment was completed over 8 d between December 2015 and January 2016. All 20 feeders were tested during the validation experiment. This experiment tested the difference between weight of feed measured by the automated feed units and that measured by external scales. The order in which feeders were tested was fully randomized for each test day.

The validation sampling procedure involved placing a cardboard box (analogue sheep) in the approach race (Figure 1c) to activate a feed event. Once the feed event had been activated, a unique RFID “test” tag was placed in front of the RFID reader to replicate the presence of a feeding animal and attach the specific RFID to the feed event. During the simulated feed event, one of four approximate sample sizes were removed by hand from the feed bin. In validation experiment 1, samples of approximately 50, 100, 200, and 400 g were removed using a volumetric approach. Different sized plastic scoops were used to collect the pellets and remove the samples from each feeder. Samples of pellets were placed in plastic bags that were sealed and the pellets later weighed on a calibrated portable weigh scales (FG-31 KBM—30- × 0.001-kg scales, A&D Co. Ltd., Korea). These external scales were calibrated using calibrated reference weights (100 and 200 g and 1 and 2 kg). After the feed sample had been removed from the feed bin, the RFID tag was removed from the read range of the automated feeder and the analogue sheep was removed from the approach race to close the feed event. This process was repeated for the same feeder until all four samples had been removed. The feed bin within each feeder was allowed to fill to the maximum meal allocation (~1,000 g) between samples. The process was then repeated at the next feeder and the order in which feeders were tested was randomized.

Each sample size was associated with an individual RFID tag. When all samples had been collected, data for the appropriate feed events were accessed via the FMS. To determine whether each feed event was allocated to the correct RFID tag, the order of the feed event creation was compared with the order of RFID tag detected and recorded by the feeding system.

### Experiment 2

The second validation experiment was completed in September 2016. Sampling was completed over 3 d: days 10, 11, and 12 of the feed intake test period (Table 1).

Feed events were activated and ended via the placement or removal of the analog sheep and RFID test tag as in experiment 1. Recognition of the RFID “test” tag for each feed event was confirmed by accessing the FMS software (via an iPad). Consistent with experiment 1, after a feed event had been activated, one of four sample sizes were removed using different-sized plastic scoops from the feed bin to simulate the consumption of pellets by a sheep. The removed samples were estimated on a volumetric basis to be approximately 50, 100, 200, or 400 g. When a sample was removed from the feed bin, it was immediately weighed on a calibrated set of portable weigh scales located at the feed intake facility (FG-31 KBM—30- × 0.001-kg scales, A&D Co. Ltd., Korea).

### Experiment 3

The final validation experiment was conducted between October 2017 and January 2018 (Table 1). Sampling from each feeder was conducted on day 1 (test 1), day 57 (test 2), and day 71 (test 3) of an associated 11-wk feeding study using sheep.

Validation sampling was completed using the same method described for experiments 1 and 2. Feed events were activated and samples of 50, 100, 200, and 400 g were collected from feeders on a volumetric basis and weighed as described in experiment 2. However, in this experiment, the creation and completion of the feed event and allocation to the correct sampling RFID tag was confirmed at the time of sample collection via a physical test of the locked entry gate and through FMS software (accessed via an iPad).

### Statistical Analysis

Analysis was conducted on each experiment separately and followed a method comparison approach, where the weight of feed manually removed from the feed bin (and weighed on the portable calibrated scales) was assigned as the control/standard value. This control value was then compared to the weight of feed that was measured by the feeding system and assigned to the feed event (with corresponding RFID tag) using the FMS.

For each validation experiment, the manual weight and feeding system weight for the same feed event/sample were compared. A paired *t*-test was used to determine the overall relative bias between the measurement methods. Bland–Altmann plots with regression were used to graphically examine and estimate the bias and limits of agreement between the methods (Bland and Altman, 1999). In addition, since each method cannot be considered to be without error, further testing of the bias was conducted by examining the geometric mean functional regression of the paired values as advocated by Ludbrook 2010. The estimate of the slope for the Bland–Altman regression was compared with 0 and the estimate of the slope for the geometric mean functional regression compared with 1 by *t*-test. CIs (95%) for all estimates of bias and slope were also determined. The linear association between the two measurement methods was examined by calculating the correlation coefficient. However, since correlations coefficients do not test the agreement between two methods and have limitations with respect to their use and interpretation in method comparisons, Lin’s concordance correlation coefficients (CCC) were calculated for each experiment (Lin, 1989). The CCC is an index of reproducibility that can be used to evaluate the agreement between paired measurements. All analyses were conducted in Genstat version 18.2 (VSN International Ltd).

## RESULTS

### Variation in Volumetric Sampling

Descriptive statistics based on the volumetric target sample weight (50, 100, 200, and 400 g) are shown in Table 2. In all validation experiments, mean sample weight collected was similar to the target sample weight; however, the SD and range between maximum and minimum weight were larger for the 200- and 400-g samples in experiment 1 than for those samples collected in experiments 2 and 3.

**Table 2.** The number of samples, mean, SD, and maximum and minimum of measured sample weights* of samples collected volumetrically from the feed bin in experiments 1, 2, and 3

Nominal test sample weight†, g	Number of test samples	Mean, g	SEM, g	SD, g	Minimum, g	Maximum, g
Experiment 1						
50	180	66.2	0.7	9.0	44	93
100	177	104.0	1.0	13.1	60	236
200	177	217.7	2.2	29.4	108	293
400	179	438.5	5.0	66.4	223	583
Experiment 2						
50	38	61.5	2.0	12.6	40	91
100	38	109.8	2.5	15.1	88	156
200	38	205.1	1.6	9.6	188	224
400	37	405.7	2.1	12.7	382	432
Experiment 3						
50	53	60.3	1.2	8.6	45	79
100	53	108.2	1.6	11.3	89	148
200	53	199.7	1.7	12.5	167	228
400	53	408.9	2.5	17.9	364	440

*Measured after collection on a separate calibrated portable scale.

†The nominal weight of the test sample based on volumetric collection method.

### Tag Detection

There were no instances when feed sampling events were assigned to an incorrect tag. During experiment 1, the order in which samples were taken was compared with the order of recorded events following the completion of the validation exercise. In experiments 2 and 3, the validation procedure involved checking the creation of each feed event and the RFID associated with the event prior to sample collection.

### Feed Weight Measurements

**Figure 2.**
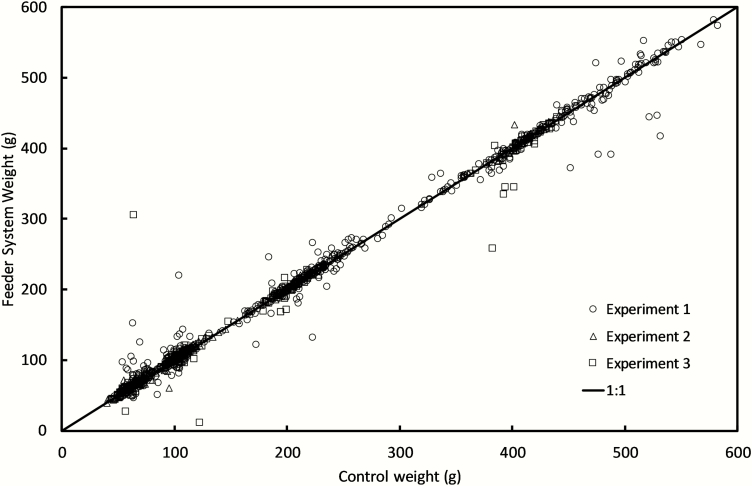
The relationship between the feeding system generated weight (g) and the weight measured on an external calibrated scale for experiments 1 (○), 2 (∆) and 3 (□). The diagonal line indicates the theoretical 1:1 relationship.

Each feeder validation experiment was conducted during a period when sheep were using the feeding system under ad libitum feeding. The results of three experiments comparing the weight of feed samples measured manually using a calibrated portable scale or measured by the feeding system are shown in Table 3. The correlation between the control method and the weights measured by the feeding system was very high at *r* = 0.99 in all three experiments. The linear association between the feeding system and control (manually weighed) measurements were consistent across all three experiments (Figure 2). The CCC estimates indicated a high degree of agreement between the control measurements and the measurements by the feeding system.

**Table 3.** The results of three experiments comparing the weight of feed removed and measured on a calibrated scale (control) and the measured weight reported by the automated feeding system (feeder)

	Experiment 1		Experiment 2		Experiment 3	
	Control	Feeder	Control	Feeder	Control	Feeder
Paired *t*-test for overall relative bias						
Number of observations	713	713	151	151	212	212
Mean, g	206.65	206.46	194.14	194.48	194.27	191.84
SD, g	150.09	148.65	132.25	132.74	134.61	133.44
SE, g	5.62	5.57	10.76	10.80	9.25	9.16
Mean bias, g ± SE	0.198 ± 0.515		0.344 ± 0.423		2.425 ± 1.515	
95% CI for mean	−0.814, 1.209		−1.181, 0.492		−0.562, 5.411	
*P*-value	0.701		0.417		0.111	
Bland–Altman plot analysis, difference = control − feeder						
Mean difference, bias	−1.805		0.383		0.723	
95% CI for mean difference	−3.525, −0.08557		−1.104, 1.869		−4.54, 5.986	
Slope difference vs. average, ± s.e.	0.0097 ± 0.0034		−0.003742 ± 0.0032		0.00881 ± 0.0114	
95% CI for slope	0.002946, 0.01645		−0.01007, 0.002587		−0.01362, 0.03125	
*P*-value	0.005		0.245		0.439	
Limits of agreement						
Lower 95% limit, slope in brackets	−11.442 (−0.01792)		−6.684 (−0.004925)		−11.835 (−0.01439)	
Upper 95% limit, slope in brackets	7.831 (0.03731)		7.449 (−0.002558)		13.280 (0.03202)	
Geometric mean functional regression, *x* = control, *y* = feeder						
Constant, mean bias	1.792 ± 0.8686		−0.3831 ± 0.7542		−0.7311 ± 2.655	
95% CI for constant	0.08703, 3.497		−1.873, 1.107		−5.966, 4.503	
Slope	0.9904 ± 0.0034		1.0037± 0.0032		0.9913 ± 0.0112	
95% CI for slope	0.9837, 0.9970		0.9974, 1.010		0.9691, 1.013	
*P*-value	0.005		0.246		0.438	
Correlation and reproducibility						
Correlation coefficient, *r*	0.9958		0.9992		0.9865	
*P*-value	<0.001		<0.001		<0.001	
Lin’s concordance correlation coefficient	0.9958		0.9992		0.9863	
95% CI	0.9951, 0.9963		0.9989, 0.9994		0.9821, 0.9895	
Bias correction factor; *C*_*b*_	1.0000		1.0000		0.9998	

**Figure 3.**
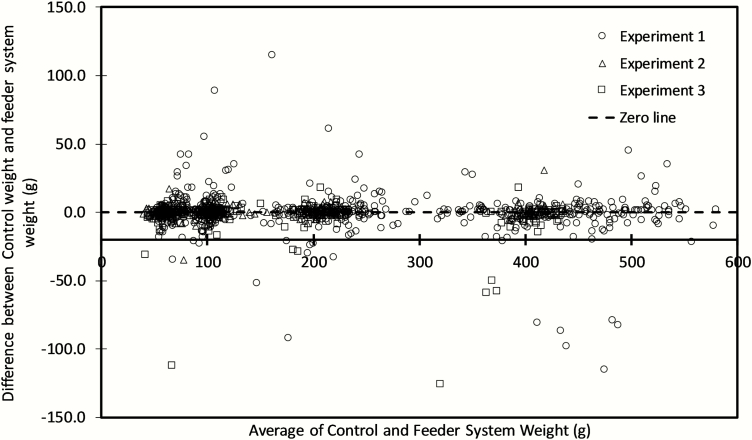
The Bland–Altman plot showing the difference between the feeding system generated weight (g) and the weight measured on an external calibrated scale for experiments 1 (○), 2 (∆), and 3 (□) plotted against the average of both measurement methods. The dashed line indicates zero difference.

For all three experiments, testing of the overall relative bias indicated that there was no general bias between the measurement methods (*P* > 0.05). Examination of the slope of the relationship between the differences in weight between the methods plotted against the average weight of the methods (Figure 3 and Table 3) showed a small but statistically significant level-dependent bias for the data collected in experiment 1. This bias was also confirmed via examination of the geometric mean functional relationship between the measurements with the slope being significantly different from unity (*P* = 0.005). Level-dependent biases were not evident for measurements undertaken in experiments 2 and 3 (*P* > 0.05).

## DISCUSSION

Errors in measuring individual animal feed intake data using automated livestock feeders may include inaccurate calibration of weigh scales, inaccurate identification of animals entering the feeders, and equipment malfunctions that may result in biases over the duration of a feed intake trial period or between feeders. All these sources of error can be managed via regular monitoring of equipment and highlight the requirements for the verification of electronic data with comparison to known standards at regular time points throughout feed intake experiments.

The feeder validation performed in these experiments evaluated four key parameters that could impact on the accuracy of feed intake results obtained using automated feeding stations and include: 1) RFID detection, 2) the correlation between the weight of the feed consumed measured by the feeding system and manually, 3) whether there was any general bias associated with the feeding system, and 4) whether there was any level-dependent bias. Experiments were conducted over 3 yr during periods when the feeding system was under full use by sheep in feeding experiments and test days were spread across the period of each of these experiments. This also allowed the assessment of the stability of the feeding system over time and between different feeders in the system. This knowledge is required to report the accuracy of automated feeding systems developed for measuring individual animal feed intake in group-housed sheep. We hypothesized that the described automated feeding system would determine individual feed events, including the logging of a feed event and the identity of the animal feeding with no overall or level-dependent bias between system-measured feed consumed and the weight of feed measured on an external scale. We also hypothesized that the performance of the feeding system would not vary over time when the feeding system was being used by sheep.

### RFID Detection

In this study, there were no instances where feed events were allocated to the incorrect RFID tag. These findings are consistent with previous reports by Chapinal et al. (2007)—100% sensitivity and 100% specificity; Chizzotti et al. (2015)—99.6% sensitivity and 99.9% specificity; Oliveira et al. (2018)—99.25% sensitivity and 98.98% specificity; and Bach et al. (2004)—99.6% sensitivity and 98.8% specificity. These authors all describe feeding systems underpinned by passive RFID transponder technology to identify individual animal feed events. RFID technology is a well-established technology used in animal identification and tracking (e.g., the Australian National Livestock Identification Scheme), automated feeding systems, and manufacturing for product tracking.

### Correlation Between Feeding System and Manually Measured Feed Intake Amounts

During all three validation experiments, we observed a high correlation (*r* = 0.99) between the feeding system-measured and manually measured sample weights. This data suggests that when recording feed intake of individual sheep over a single feed event, the error associated with the amount of feed intake measured (g) are small in relation to the variation across samples. These high correlations are consistent with comparable automated livestock feeding systems (Chapinal et al., 2007; Oliveira et al., 2018). Oliveira et al. (2018) reported a high correlation between the feed intake determined by an Intergado automated feed intake system and manual weighed feed intake samples (*r* = 0.986). Chapinal et al. (2007) also reported high correlations between system-generated feed intake and manually recorded feed intake (*r* = 0.99) in a feed system used for cattle. However, while correlation coefficients provide a measure of the linear association between two measures, they do not provide a measurement of agreement or reproducibility. In our analysis of these experiments, the calculations of the CCC for each experiment indicates a high degree of agreement (CCC = 0.99) between measures conducted using the control method and the feeder management system.

### Overall Relative Bias Between FMS and Manually Measured Feed Intake Amounts

There were no statistically significant overall biases between the feeding system-recorded feed amount and the manually recorded feed amount (control method) in three experiments conducted across 3 yr when the feeding system was being used with sheep in feeding experiments. These results demonstrate the stability of the feeding system over time and between feeders. Previous studies by Chapinal et al. (2007), DeVries et al. (2003), Chizzotti et al. (2015), or Bach et al. (2004) did not provide a temporal assessment of the performance of their described feeding systems under test conditions. However, Faltys et al. (2014) determined the performance of automated pig feeders during a 126-d feed intake study by comparing the amount of feed metered out and consumed by animals to the weight of feed left in the feed hopper weighed manually. This was performed every 7 d throughout the trial. When the weight of system-recorded feed differed from manually measured feed weights by greater than or less than 4%, the weigh scales on the feeder trough were recalibrated. These criteria were met in week 1 for both feeders tested and in week 9 for one of the two feeders tested in that study. In our experiments, the maintenance protocol involved the weekly checking and adjustment of scales to the zero-tare weight when there was no feed in the feed bin.

The variation in sample weights collected on a volumetric basis highlights the need for a feeder that is able to weigh dispensed feed as opposed to relying on a volumetric measurement. When the weight of samples collected volumetrically was considered based on the target sample weight (Table 2), there was a considerable variation in the samples collected at all target sample weights. In experiment 1, samples collected to be 400 g (by volume) ranged from 223 to 583 g. Variation in sample weights was smaller for the 200- and 400-g samples collected in experiments 2 and 3. This is attributed to some extent to methodology, whereby the same operator was used consistently throughout experiments 2 and 3, while a number of different operators were used in experiment 1, perhaps resulting in less-consistent sample collection. The variation associated with volumetric sampling may be particularly important when the size and weight of the pellets are large and the meal size is small. For example, individual pellets used in this experiment, although compressed to 9 mm in diameter were observed to vary between ~0.6 and 2.07 g for an individual pellet (mean 1.5 g), as well as varying in density, size, and shape due to the manufacturing process. This will impact on the weight of a given volume of pellets. Volumetric feeders should be calibrated regularly to ensure that they are delivering target weights of feed, although variation may be smaller in machine-driven systems compared with human sampling.

### Level-Dependent Bias Between the Feeding System and Manually Measured Feed Intake Amounts

A statistically significant level-dependent bias was detected in experiment 1. This experiment contained several major outliers in the difference between the weight measurement of the two methods. Values were considered to be major outliers when the difference between control and feeding system-measured weights was greater than 50 g (approximately 3–4 times the SD of the differences). The impact of these 12 outlier observations was assessed by their removal from data for analysis. This resulted in the tests for level-dependent bias examining the slope of geometric mean function regression (0.9997 ± 0.0021) and the slope of the difference versus average regression (0.0003 ± 0.0021) becoming nonsignificant (*P* = 0.885). These outliers were traced back to human procedural errors that occurred during the conduct of the testing. These procedural errors resulted in incorrect weight data being assigned to the feed event in the feeding system in comparison to the weight of the sample collected. The errors were, however, not the result of a feeding system error or incorrect RFID reading. In experiment 3, a small number of outlier measurements were recorded for a single feeder that was known to be malfunctioning and in need of repair.

Even with the inclusion of the outliers, the bias detected in experiment 1 would only result in a 4.3 g reduction across the range of 50–500 g in a feed amount consumed. This would result in an overestimation of intake at 50 by 1.3 g and underestimating the intake by 3.0 at 500 g. This experiment had no overall bias.

The meal size of sheep in the ad libitum feeding experiments conducted typically average 162–243 g per meal and range from 64 to 558 g per meal (Muir et al., 2018a). However, meal size and meal number are interrelated with total feed intake per day such that animals consuming smaller meals tend to eat more meals per day (Muir et al., 2018a). Meal number may vary between 2.4 and 31 meals per day and average approximately 10 meals per day. These results indicate that level-dependent biases in the order of that detected in experiment 1 could overestimate daily intake by about 40 g/d for animals consuming 30 meals at around 50 g per meal and underestimate feed intake by about 18 g/d for sheep consuming six meals of around 500 g per meal. Analysis of our experiment 1 data with the outliers removed resulted in there being no level-dependent bias across all experiments and we believe that this is the most accurate representation of our feeder system in practice. Nevertheless, the results provide context about the relative importance that level-dependent biases and/or overall biases may play in feed intake measurement, particularly, when the number of meals per day is high. To our knowledge, no other studies have examined whether a level-dependent (meal size) bias exists in their systems. The results reinforce the need to have procedures in place that detect and correct potential drift of weigh scales over the range of measurements being taken during feed intake testing.

The change in methodology between experiment 1 and experiments 2 and 3, whereby the start and end of a feed event were confirmed by checking the FMS display at the time of sampling, was due to the possible procedural errors that resulted in the outliers detected in experiment 1. Ensuring that the event had closed before starting the next event better reflects the normal use of the feeders by sheep, where the electronic gate lock will not open for access until the absence of a sheep is detected by the photoelectric proximity detector and the previous event is closed. These changes in the procedure resolved the issue for experiments 2 and 3.

We conclude that the automated feeding system used in this study can accurately identify and measure the meal amounts of individual sheep when housed in groups. The data collected with the system demonstrates no major biases over extended feeding periods and multiple experiments compared to conventional measurements. We consider that this makes the system suitable for the measurement of feed intake of individual sheep in group housing. The described system provided an accurate means by which various elements of a sheep’s feeding behavior can be ascertained; they include 1) the creation of a feed event when animals enter a feeder, 2) the accurate linking of a feed event to an RFID, and 3) the meal amount of individual feed events. Since the concordance correlation coefficients with the control measurement were very high, and there is little bias, the cumulative total of feed consumed will provide an accurate measure of daily feed intake and average feed intake over extended periods.
